# TRPM7-Mediated Calcium Transport in HAT-7 Ameloblasts

**DOI:** 10.3390/ijms22083992

**Published:** 2021-04-13

**Authors:** Kristóf Kádár, Viktória Juhász, Anna Földes, Róbert Rácz, Yan Zhang, Heike Löchli, Erzsébet Kató, László Köles, Martin C. Steward, Pamela DenBesten, Gábor Varga, Ákos Zsembery

**Affiliations:** 1 Department of Oral Biology, Semmelweis University, H-1089 Budapest, Hungary; kadar.kristof@dent.semmelweis-univ.hu (K.K.); juhasz.viktoria1994@gmail.com (V.J.); anna.foldes@gmail.com (A.F.); racz62@gmail.com (R.R.); heike.loechli@gmail.com (H.L.); martin.steward@me.com (M.C.S.); varga.gabor@dent.semmelweis-univ.hu (G.V.); 2 Department of Orofacial Science, University of California, San Francisco, CA 94143, USA; Yan.Zhang2@ucsf.edu (Y.Z.); Pamela.DenBesten@ucsf.edu (P.D.); 3 Department of Pharmacology and Pharmacotherapy, Semmelweis University, H-1089 Budapest, Hungary; kato.erzsebet@med.semmelweis-univ.hu (E.K.); koles.laszlo@med.semmelweis-univ.hu (L.K.); 4 School of Medical Sciences, University of Manchester, Manchester M13 9PL, UK

**Keywords:** TRPM7 channel protein, enamel, pH, calcium entry, magnesium, amelogenesis, store-operated calcium entry

## Abstract

TRPM7 plays an important role in cellular Ca^2+^, Zn^2+^ and Mg^2+^ homeostasis. TRPM7 channels are abundantly expressed in ameloblasts and, in the absence of TRPM7, dental enamel is hypomineralized. The potential role of TRPM7 channels in Ca^2+^ transport during amelogenesis was investigated in the HAT-7 rat ameloblast cell line. The cells showed strong TRPM7 mRNA and protein expression. Characteristic TRPM7 transmembrane currents were observed, which increased in the absence of intracellular Mg^2+^ ([Mg^2+^]_i_), were reduced by elevated [Mg^2+^]_i_, and were inhibited by the TRPM7 inhibitors NS8593 and FTY720. Mibefradil evoked similar currents, which were suppressed by elevated [Mg^2+^]_i_, reducing extracellular pH stimulated transmembrane currents, which were inhibited by FTY720. Naltriben and mibefradil both evoked Ca^2+^ influx, which was further enhanced by the acidic intracellular conditions. The SOCE inhibitor BTP2 blocked Ca^2+^ entry induced by naltriben but not by mibefradil. Thus, in HAT-7 cells, TRPM7 may serves both as a potential modulator of Orai-dependent Ca^2+^ uptake and as an independent Ca^2+^ entry pathway sensitive to pH. Therefore, TRPM7 may contribute directly to transepithelial Ca^2+^ transport in amelogenesis.

## 1. Introduction

Ameloblasts are highly specialized epithelial cells with a key role in the mineralization of dental enamel. In this role, maturation ameloblasts mediate transepithelial secretion of Ca^2+^ from the blood to the site of mineralization, close to the apical surface of the cells [1,2]. Key to the function of maturation ameloblasts is their modulation of the acidity in the enamel-forming space, periodically cycling the extracellular pH between pH 6.2 and 7.2 while alternating between ruffle-ended and smooth-ended morphologies [1,3]. These pH changes are required both for the transport of dissolved Ca^2+^ and phosphate ions to the mineralization space, and for the subsequent precipitation of these ions into hydroxyapatite crystals.

The apical membrane secretion of Ca^2+^ into the mineralization space is achieved by multiple calcium transporters, including the plasma membrane Ca^2+^-ATPase (PMCA) and both potassium-dependent and -independent Na^+^/Ca^2+^ exchangers [4,5]. The basolateral uptake mechanisms for Ca^2+^ are less well understood, but are largely attributed to store-operated Ca^2+^ entry (SOCE) channels [6,7,8,9]. However, the large amount of calcium that must be transported to the highly mineralized enamel matrix suggests the possibility of additional mechanisms, such as calcium uptake via the divalent-cation-permeable TRPM7 channel. The potential role of TRPM7 in cellular calcium transport has been shown in other cell types, for example in the regulation of human leukemia cells [10]. TRPM7 channels are also known to mediate Ca^2+^ uptake in eggs at fertilization [11] and to play a pivotal role in intestinal Ca^2+^ absorption [12]. In addition, TRPM7 ion channels exhibit high Zn^2+^ conductivity and regulate cytosolic and nuclear Zn^2+^ concentrations [13] TRPM7 proteins are not only involved in Ca^2+^/Mg^2+^/Zn^2+^ homeostasis but also regulate matrix metalloproteinase (MMP)-13 gene expression and alkaline phosphatase activity in chondrocytes [14].

TRPM7 is ubiquitously expressed in various organs, and relatively high mRNA expression levels have been detected in teeth [15]. TRPM7 protein expression has been shown in various stages of ameloblast development, and the intensity of the immunostaining increases considerably during the maturation stage [16]. During the early stages of the ameloblast differentiation, the TRPM7 kinase domain plays a pivotal role in the activation of bone morphogenic protein (BMP) signaling via phosphorylation of cAMP response element binding (CREB) protein [15]. The deletion of the TRPM7 kinase domain causes hypomineralized dental enamel with reduced volume [15,16]. Lacruz and colleagues have recently suggested that the TRPM7 channel may not itself provide a Ca^2+^ entry pathway in ameloblasts, but acts instead as a positive modulator of SOCE by potentiating Orai-dependent Ca^2+^ uptake [17].

Studies performed in native and heterologous expression systems indicate that TRPM7-mediated currents are regulated both by the intracellular Mg^2+^ concentration ([Mg^2+^]_i_) and by pH. The depletion of cellular Mg^2+^ is known to activate TRPM7-mediated currents [18], and both extra- and intracellular protons have been shown to inhibit these currents in the absence of divalent cations [19,20]. In the presence of divalent cations, however, H^+^ may have a net stimulatory effect [20]. Changes in extracellular [Mg^2+^] and pH during enamel formation might therefore be expected to regulate TRPM7-dependent Ca^2+^ and Mg^2+^ uptake into the ameloblasts.

We therefore hypothesize that TRPM7 has significant roles in ameloblast cells, not only as a regulator of SOCE, but also as a divalent cation channel, and that these functions may be modulated by Mg^2+^ and pH changes. To explore this possibility, we have used the HAT-7 cell line, derived from rat incisor cervical loop [21], to investigate the expression, activity and pH sensitivity of TRPM7 channels. HAT-7 cells exhibit ameloblast-like characteristics, including the expression of amelogenin, ameloblastin, and the maturation-stage ameloblast proteins kallikrein-4 and amelotin [21]. Following their initial characterization, HAT-7 cells have been used in a large number of studies to investigate ameloblast function [22,23,24,25,26]. In particular, we have shown previously that the HAT-7 cell line provides a good experimental model for investigating ion transport mechanisms [3,27,28,29].

In the present study, we have measured mRNA and protein expression, ion currents evoked by TRPM7-specific activators and inhibitors during manipulation of extra- and intracellular [Mg^2+^] and pH, and the corresponding changes in intracellular Ca^2+^ concentration ([Ca^2+^]_i_) and pH (pH_i_).

## 2. Results

### 2.1. TRPM7 Is Expressed in HAT-7 Cells and in Mouse Ameloblasts

**Figure 1:**
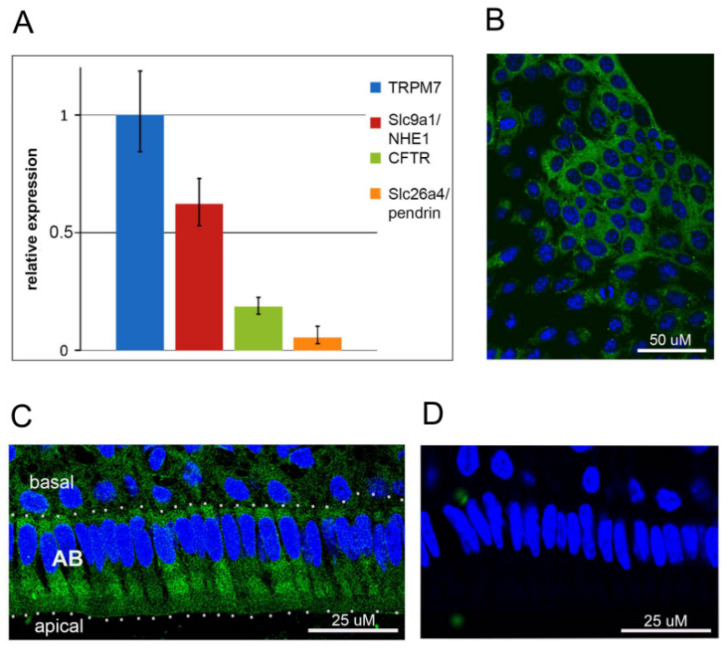
Expression of TRPM7 in HAT-7 cells and mouse incisor ameloblasts. (**A**) RT-qPCR analysis of the mRNA expression levels of TRPM7, Slc9a1/NHE1, CFTR and Slc26a4/pendrin normalized to mean TRPM7 expression in HAT-7 cells. (**B**,**C**) Immunolocalization of TRPM7 protein (green) in HAT-7 cells grown on glass coverslips (**B**) and in maturation-stage ameloblasts (AB) from mouse incisor (**C**). (**D**) Negative control staining using nonspecific rabbit IgG in maturation-stage ameloblasts. Nuclei, blue.

Quantitative gene expression analysis showed that TRPM7 mRNA was highly amplified in HAT-7 cells, relative to other amelogenesis-related ion transporters and channels, including NHE1, CFTR and pendrin (Figure 1A). The presence of the TRPM7 protein in HAT-7 cells was shown by immunostaining with a polyclonal antibody against the intracellular C-terminal of human TRPM7 (Figure 1B). In vivo, TRPM7 followed a similar, largely cytoplasmic, distribution in mouse maturation-stage ameloblasts (Figure 1C). The negative controls showed no positive immunostaining for TRPM7 (Figure 1D).

### 2.2. HAT-7 Cells Exhibit TRPM7-Like Ion Currents

**Figure 2:**
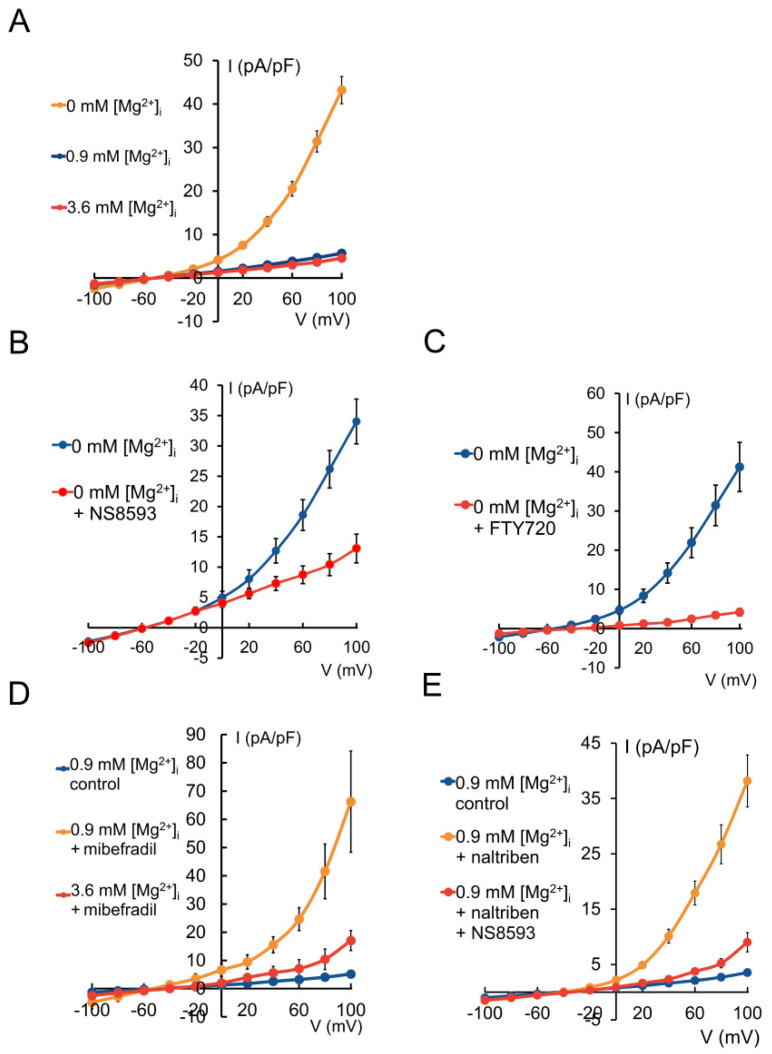
Whole-cell patch-clamp recordings of TRPM7 currents in HAT-7 cells. (**A**) TRPM7-related currents at different intracellular Mg^2+^ concentrations (*n* = 14). Effects of inhibitors on TRPM7 currents induced by a nominally Mg^2+^-free intracellular solution: (**B**) NS8593 (20 µM, *n* = 5) and (**C**) FTY720 (2 µM, *n* = 5). Effects of TRPM7 activators: (**D**) currents evoked by mibefradil (50 µM) in the presence of 0.9 mM (*n* = 5) and 3.6 mM [Mg^2+^]_i_ (*n* = 4); (**E**) currents evoked by naltriben (50 µM) in the absence and presence of NS8593 (50 µM, *n* = 4) at physiologic [Mg^2+^]. Data are presented as means ± SEM.

The characteristic electrophysiological signature of the TRPM7 channel is an outwardly rectifying cation current—usually recorded as a Cs^+^ current—that is stimulated by depletion of intracellular Mg^2+^ [18,30]. Whole-cell patch-clamp recordings from HAT-7 cells (Figure 2A), using bath and pipette solutions that suppress other currents, showed a small outwardly rectifying current (4.7 ± 0.14 pA/pF at +80 mV, *n* = 14) when the intracellular solution contained a physiological concentration of Mg^2+^ (0.9 mM). When the intracellular Mg^2+^ was increased from 0.9 to 3.6 mM, there was a small but significant decrease in the current (to 3.6 ± 0.2 pA/pF at +80 mV, *n* = 14, *p* < 0.05). However, when Mg^2+^ was removed from the intracellular solution, there was a dramatic, six-fold increase in the current (to 29.8 ± 2.4 pA/pF at +80 mV, *n* = 14, *p* < 0.0001).

Further confirmation that this current was due to TRPM7 was obtained by means of two established TRPM7 inhibitors, NS8593 [31] and FTY720 [17], which were used to probe the stimulated currents elicited by intracellular Mg^2+^ depletion (Figure 2B,C). In the nominal absence of intracellular Mg^2+^, 20 µM NS8593 reduced the current at +80 mV by 60% (from 26.1 ± 3.0 to 10.4 ± 1.8 pA/pF, *n* = 5, *p* < 0.05), and 2 µM FTY720 reduced it by 90% (from 31.4 ± 5.2 to 3.4 ±0.8 pA/pF, *n* = 5, *p* < 0.001).

The stimulatory effects of the two pharmacological activators of TRPM7, mibefradil [32] and naltriben [33], were also examined by whole-cell current recording from HAT-7 cells (Figure 2D,E). When intracellular Mg^2+^ was at a physiological concentration (0.9 mM), extracellular mibefradil (50 µM) caused a 10-fold increase in the outward current from 4.0 ± 0.4 to 41.5 ± 9.7 pA/pF at +80 mV (*n* = 5, *p* < 0.05) (Figure 2D). Elevated intracellular Mg^2+^ (3.6 mM) reduced the mibefradil-stimulated current by 75% to 10.4 ± 3.6 pA/pF (*n* = 4, *p* < 0.05).

Stimulation with 50 µM naltriben also significantly increased the outward current at +80 mV, from 2.7 ± 0.3 to 26.7 ± 3.5 pA/pF (*n* = 4, *p* < 0.0001) (Figure 2E). This increase was largely reversed by the TRPM7 inhibitor NS8593 (20 µM), which reduced the current by 80% to 5.2 ± 0.8 pA/pF (*n* = 4, *p* < 0.05).

### 2.3. TRPM7 Activators Stimulate Ca^2+^ Influx in HAT-7 Cells

Although the electrophysiology of TRPM7 channels is usually investigated, as here, by using Cs^+^ as the conducting ion, these channels are highly permeable to divalent cations. We therefore next examined whether they might contribute directly to Ca^2+^ uptake into HAT-7 cells, in addition to their role in modulating the SOCE pathway [34].

**Figure 3:**
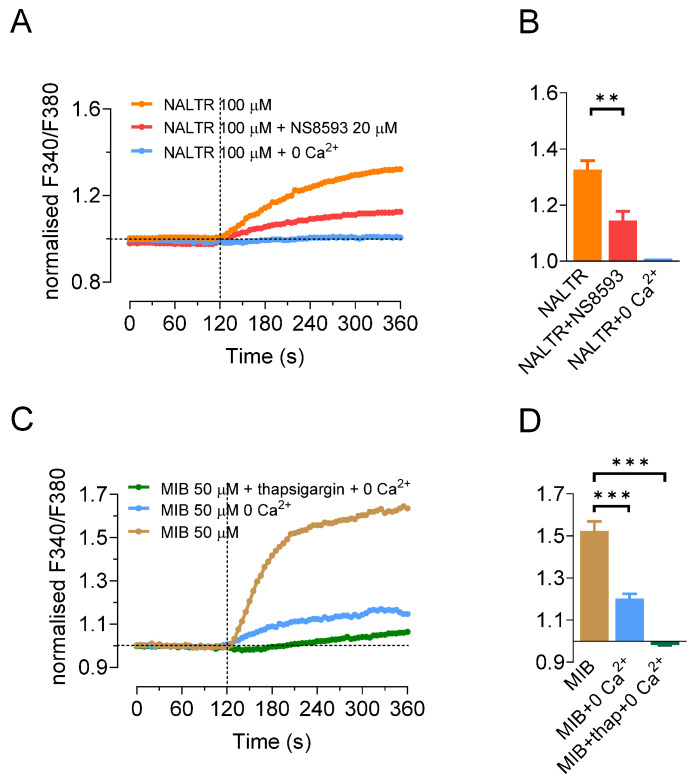
Changes in intracellular Ca^2+^ in response to TRPM7-specific activators and inhibitors. (**A**,**B**) Changes in [Ca^2+^]_i_ evoked by naltriben (100 µM, applied at 120 s) in the presence (*n* = 9) and absence of extracellular Ca^2+^, and in the presence of 20 µM NS8593 (applied at 0 s, *n* = 5). (**C**,**D**) Changes in [Ca^2+^]_i_ evoked by mibefradil (50 µM, applied at 120 s) in the presence (*n* = 12) and absence (*n* = 6) of extracellular Ca^2+^, and following pretreatment with 100 nM thapsigargin (*n* = 4). Ca^2+^ was present in the bath solution unless the legend states otherwise. Data are presented as representative traces of changes in fura-2 fluorescence ratio normalized to baseline, and as mean peak values ± SEM (** *p* < 0.01, *** *p* < 0.001).

Changes in the intracellular Ca^2+^ were measured using fluorescence imaging of the Ca^2+^-sensitive fluoroprobe fura-2. The cells were bathed in a nominally Mg^2+^-free extracellular solution in order to minimize competition with Ca^2+^. The TRPM7 activator naltriben (100 µM) elicited a sustained and reversible 32.6 ± 3.3% increase in [Ca^2+^]_i_ (*n* = 9, *p* < 0.05) (Figure 3A,B). This increase was completely abolished in a nominally Ca^2+^-free bath solution (Figure 3A,B) confirming that the increase in [Ca^2+^]_i_ was due to Ca^2+^ influx from the extracellular space. The naltriben-induced increase in [Ca^2+^]_i_ was reduced by 56% (*n* = 5, *p* < 0.01) by the TRPM7 inhibitor NS8593 (20 µM) (Figure 3A,B).

Stimulation with 50 µM mibefradil increased [Ca^2+^]_i_ by 52 ± 5% (*n* = 12, *p* < 0.05) (Figure 3C,D). Unlike the experiments with naltriben, the [Ca^2+^]_i_ response was only reduced by approximately 60% to 20 ± 2% (*n* = 6, *p* < 0.004) in the nominally Ca^2+^-free bath solution (Figure 3C,D). However, pretreatment with the SERCA inhibitor thapsigargin (100 nM), to deplete intracellular Ca^2+^ stores, completely abolished the increase in [Ca^2+^]_i_ evoked by mibefradil, even resulting in a transient decrease of 1.8 ± 0.2% below baseline (*n* = 4, *p* < 0.05) (Figure 3C,D).

### 2.4. SOCE Blocker BTP2 Inhibits Ca^2+^ Influx Stimulated by Naltriben but Not Mibefradil

**Figure 4:**
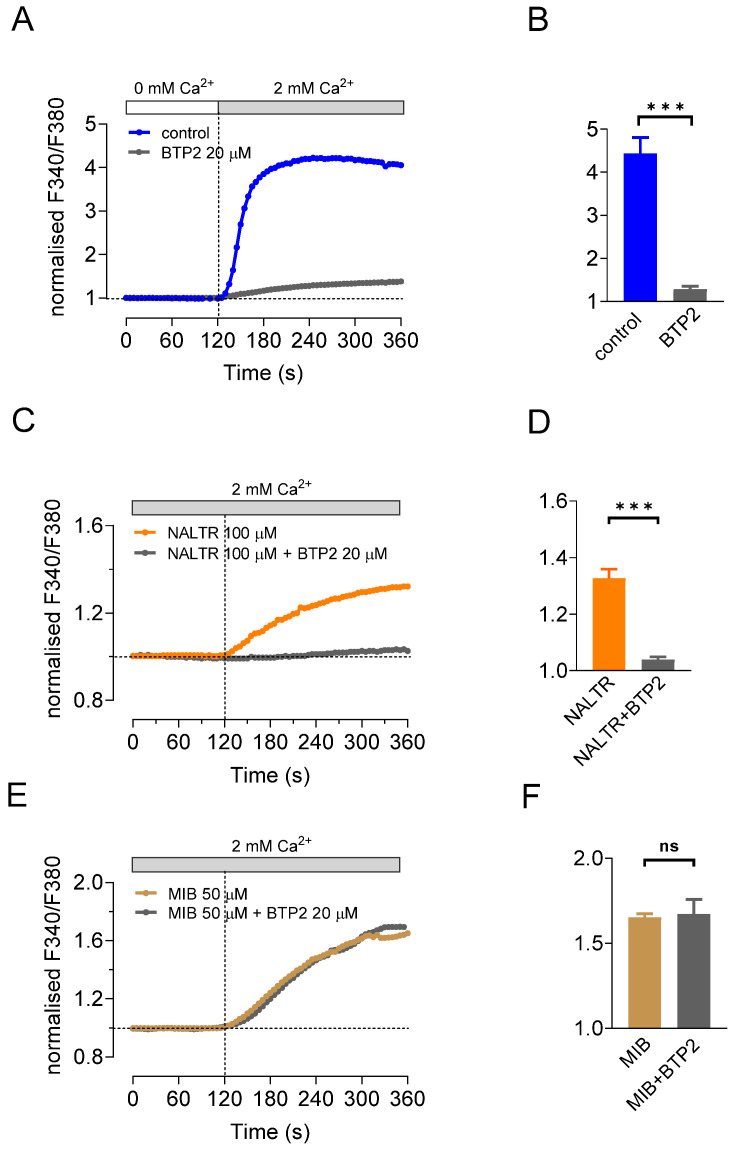
Changes in intracellular Ca^2+^ in response to TRPM7 activators and the SOCE inhibitor BTP2. Changes in [Ca^2+^]_i_ elicited by restoration of extracellular Ca^2+^ in ER store-depleted cells following thapsigargin pretreatment (100 nM, 20 min) in Ca^2+^-free bath solution, in the presence (*n* = 3) and absence of BTP2 (20 µM, applied at 0 s, *n* = 3) (**A**,**B**). Changes in [Ca^2+^]_i_ evoked by naltriben (100 µM, *n* = 4) (**C**,**D**) and mibefradil (50 µM, *n* = 5) (**E**,**F**) applied at 120 s in the presence and absence of BTP2 (20 µM, applied at 0 s). Data are presented as representative traces of changes in fura-2 fluorescence ratio normalized to baseline, and as mean peak values ± SEM (*** *p* < 0.001).

To investigate the contribution of SOCE to the Ca^2+^ influx evoked by stimulation of TRPM7 (as proposed by Souza Bonfim et al. [34]), responses to the two TRPM7 activators were also measured in the presence of a known SOCE blocker, BTP2 [35]. First, HAT-7 cells were pretreated with thapsigargin in the absence of extracellular Ca^2+^ to deplete the ER stores, and the effect of Ca^2+^ readdition was then tested in the presence and absence of BTP2. Pretreatment with BTP2 (20 µM, 45 min), and its continued presence in the bath solution, effectively blocked the SOCE-related Ca^2+^ influx in unstimulated HAT-7 cells (92% inhibition; *n* = 3, *p* < 0.001) (Figure 4A,B).

Next, we tested the effect of BTP2 on the Ca^2+^ influxes evoked by naltriben (100 µM) and mibefradil (50 µM) without prior depletion of the intracellular stores. The stimulation of Ca^2+^ influx by naltriben (in normal extracellular Ca^2+^) was almost totally blocked by BTP2 (88% inhibition; *n* = 4, *p* < 0.001) (Figure 4C,D). In contrast, the stimulation of Ca^2+^ influx by mibefradil, under the same conditions, was completely unaffected by BTP2 (*n* = 5) (Figure 4E,F). This suggests that the influx of Ca^2+^ evoked by mibefradil was not mediated by the SOCE pathway, and is more likely to be mediated by the TRPM7 conductance itself.

### 2.5. TRPM7 Currents and Ca^2+^ Influx Are pH Sensitive

**Figure 5:**
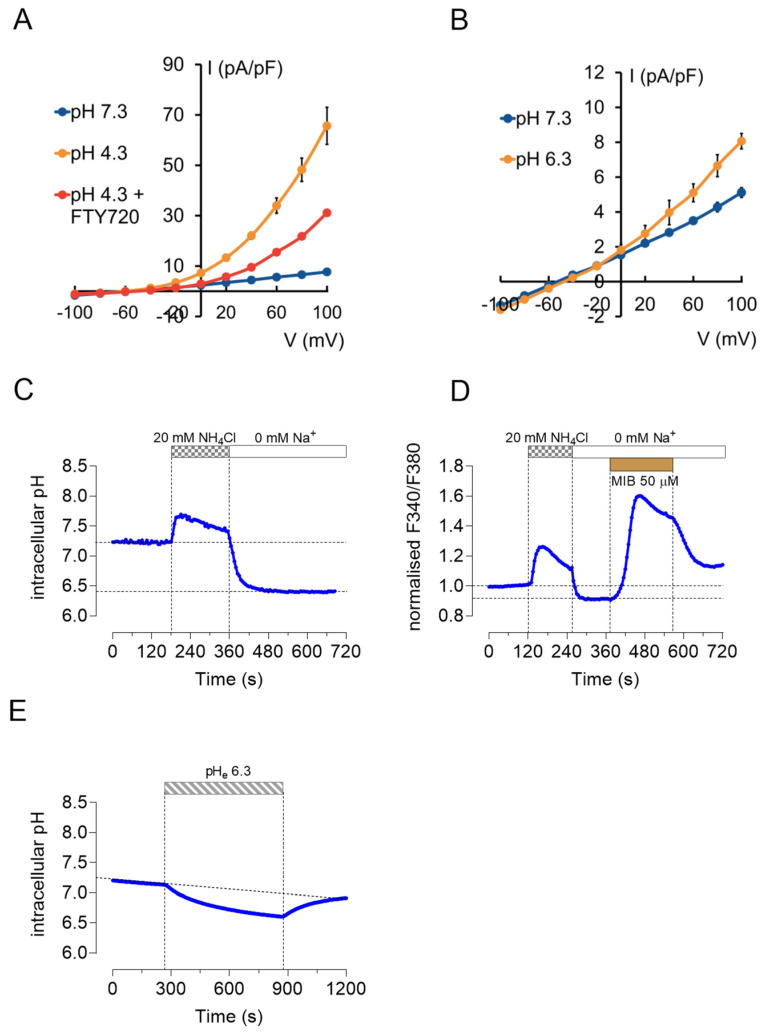
Effects of extra- and intracellular acidification on TRPM7-related ion currents and Ca^2+^ influx. Effects of (**A**) significant (pH 4.3, *n* = 4) and (**B**) modest (pH 6.3, *n* = 4) extracellular acidification on TRPM7-related currents in the presence and absence of FTY720 (2 µM, *n* = 5). (**C**) Intracellular pH changes in response to an NH_4_Cl pulse (20 mM) followed by an Na^+^-free bath solution measured by microfluorometry (representative trace). (**D**) Mibefradil-induced Ca^2+^ entry in acidic intracellular conditions measured by Ca^2+^ imaging (representative trace; data are presented as changes in fura-2 fluorescence ratio normalized to baseline). (**E**) Effect of extracellular acidification (pH 6.3) on intracellular pH measured by microfluorometry (representative trace).

In whole-cell current recordings from HAT-7 cells (Figure 5A), extracellular acidification to pH 4.3 caused a 7-fold increase in the outward current from 6.6 ± 0.7 to 48.2 ± 4.6 pA/pF at +80 mV (*n* = 4 *p* < 0.05). This proton-stimulated current was approximately halved, to 21.8 ± 1.3 pA/pF (*n* = 4, *p* < 0.05 compared to pH 4.3), by the TRPM7 inhibitor FTY720 (2 µM). A more modest extracellular acidification, from pH 7.3 to 6.3, as occurs cyclically at the apical membrane of maturation ameloblasts in vivo, also caused a small but significant increase in the outward current from 4.3 ± 0.2 to 6.6 ± 0.6 pA/pF at +80 mV (*n* = 5, *p* < 0.05) (Figure 5B).

The acidification of the cytosol unfortunately resulted in unstable membrane–glass seals, so we were unable to use whole-cell recording to examine the effects of the changes in pH_i_ on the TRPM7 currents. However, we were able to investigate the pH_i_ sensitivity of the TRPM7-mediated Ca^2+^ influx by applying the ammonium prepulse method, which is widely used to study cellular pH homeostasis.

The application of NH_4_Cl (20 mM) for 2 min caused a transient increase in pH_i_ followed by a rapid acidification on its withdrawal, as shown by fluorometric measurement of BCECF fluorescence in HAT-7 cells (Figure 5C). Following the NH_4_^+^ pulse, extracellular Na^+^ was substituted with the impermeant organic cation NMDG^+^ to inhibit proton extrusion by Na^+^/H^+^ exchange, resulting in a sustained acidification of the intracellular milieu to approximately pH 6.4 (Figure 5C). During the NH_4_^+^-evoked alkalization, [Ca^2+^]_i_ initially increased by 35 ± 4.2% (*n* = 9, *p* < 0.05) in parallel experiments under identical conditions (Figure 5D). This increase was abolished in a nominally Ca^2+^-free bath solution (data not shown). The subsequent sustained intracellular acidification, following withdrawal of NH_4_^+^ and substitution of Na^+^, caused a small but significant decrease in [Ca^2+^]_i_ by 12 ± 2% (*n* = 6, *p* < 0.05). However, applied under these conditions, both mibefradil (50 μM) and naltriben (100 μM) significantly enhanced the TRPM7-mediated Ca^2+^ influx compared to what was observed at normal pH_i_. The rise in [Ca^2+^]_i_ evoked by mibefradil increased from 43 ± 6% at normal pH_i_ to 78 ± 3.9% during the intracellular acidification (a percentage increase of 81%, *n* = 6, *p* < 0.05) (Figure 5D). Likewise, the rise in [Ca^2+^]_i_ evoked by naltriben increased by 46% ± 16% under similar conditions (*n* = 5, *p* < 0.05), indicating a marked stimulatory effect of intracellular protons on the activation of TRPM7. In this context it is interesting to note that a reduction in extracellular pH from 7.3 to 6.3, as applied in the whole-cell current recordings shown in Figure 5B, also caused a moderate and reversible acidification of the cytosol by 0.38 ± 0.032 pH units after 10 min (*n* = 6, *p* < 0.05), as measured by BCECF fluorescence (Figure 5E).

Taken together these results indicate the presence of functional TRPM7 channels in HAT-7 ameloblast cells. They also suggest that TRPM7 channels, in addition to their role in modulating SOCE, may serve as a Ca^2+^ uptake pathway sensitive to changes in both extra- and intracellular pH.

## 3. Discussion

In this study we have shown that TRPM7 channels are expressed abundantly in HAT-7 cells, both at mRNA and protein levels. This finding is not surprising in light of the previous work showing that TRPM7 expression is very high in ameloblasts and odontoblasts as compared with other organs [15], and that TRPM7 expression in ameloblasts increases progressively with differentiation towards the maturation ameloblast phenotype [16]. Since TRPM7 has recently been included in the class of functional intracellular TRP channels [13] the abundant cytoplasmic localization is not surprising. However, the intensity of the labelling makes it difficult to determine the plasma membrane localization of the channels.

In our experiments, intracellular Mg^2+^ depletion in HAT-7 cells elicited outward currents that were inhibited by the known TRPM7 antagonists NS8593 [31] and FTY720 [17]. These properties are characteristic of TRPM7 [36,37] and they therefore indicate that HAT-7 cells express functionally active TRPM7 channels. The pharmacological activators of TRPM7, naltriben [33] and mibefradil [32], evoked similar currents, which were considerably reduced by NS8593 and by elevated [Mg^2+^]_i_. It is important to note that in our experiments the presence of divalent cations in the extracellular solution prevented the activation of the inward currents that have been observed by others [36,37] in divalent cation-free extracellular conditions.

The measurement of the reversal potentials is difficult under these conditions because the current is nonlinear and has a shallow slope around E_rev_. In our experiments we used low intracellular EGTA concentrations so that Ca^2+^ remained largely unbuffered, possibly explaining the shift of E_rev_ to more hyperpolarized potentials. Although we did not evaluate the reasons for this shift, we speculate that elevated [Ca^2+^]_i_ may cause small increases in Ca^2+^-activated K^+^ channel activity. Since our bath solution contained only 2.8 mM K^+^, we assume that the negative E_rev_ values are mainly due to some Cs^+^ permeability of the K^+^ channels [38] rather than K^+^ currents.

Ca^2+^ secretion into the enamel space by ameloblasts requires Ca^2+^ uptake at the basolateral membrane to balance Ca^2+^ efflux across the apical membrane. As seen in other cell types [36,39], the uptake of extracellular Ca^2+^ by HAT-7 cells was significantly stimulated by the TRPM7 activators. Naltriben evoked a marked increase in [Ca^2+^]_i_ that was completely abolished in a nominally Ca^2+^-free environment, indicating that the TRPM7 channels can contribute to Ca^2+^ influx into HAT-7 cells. However, the SOCE inhibitor BTP2 strongly blocked the naltriben-induced Ca^2+^ entry, suggesting a pivotal role for Orai channels in these processes. This observation is in agreement with a previous report by Souza Bomfim et al. [34].

These findings are also consistent with previous in vivo studies showing the differential roles of the TRPM7 channel and kinase domains in enamel and bone mineralization [16]. The Trpm7^-/+^ mouse model used in the study by Nakano et al. was generated by replacing exons 32–36 of the Trpm7 gene with the Neo gene cassette [40]. These mice have reduced Mg^2+^ transport to bone [40] and enamel extracellular matrix [16], indicating altered TRPM7 channel activity. Enamel and bone hypomineralization in this mouse model was far more severe than in the mouse model with a point mutation in the TRPM7 kinase domain with intact channel activity [15]. These in vivo models support our findings that while TRPM7 clearly has a role in SOCE related Ca^2+^ transport, Ca^2+^ is also transported by way of the TRPM7 channel.

Mibefradil, which is also a known voltage-gated Ca^2+^ channel blocker, evoked a similar increase in [Ca^2+^]_i_. Importantly, the mibefradil-induced Ca^2+^ entry was not inhibited by BTP2, which implies that TRPM7 channels may provide a Ca^2+^ uptake pathway independent of SOCE. Our data suggest that mibefradil not only stimulated Ca^2+^ entry from the extracellular space, but also induced Ca^2+^ release from intracellular stores. Furthermore, we observed that mibefradil slightly reduced [Ca^2+^]_i_ in cells pretreated with thapsigargin. This is consistent with recent studies showing that mibefradil reduces the Ca^2+^ leak from the ER following SERCA inhibition in endothelial and HK-2 epithelial cells [41].

There are believed to be two types of TRPM7 activators: type 1 (such as naltriben), which activate the channel regardless of intracellular Mg^2+^ concentration, and type 2 (such as mibefradil), which activate the channel at low intracellular Mg^2+^ concentration [42]. It is possible that if intracellular Mg^2+^ levels are low, TRPM7 functions as a divalent-cation-permeable pathway (mimicked by mibefradil activation). Faouzi et al. have pointed out that some Ca^2+^ influx through TRPM7 is essential for the maintenance of ER Ca^2+^ stores in resting lymphocytes, and for the refilling of the stores after a Ca^2+^ signalling event [43]. However, they also support the idea that the kinase activity of TRPM7 plays an important role in the regulation of the SOCE in lymphocytes, as seems to be the case in ameloblasts. Nonetheless, further studies are required to discriminate between the mechanisms by which naltriben and mibefradil act on TRPM7 channels.

As the enamel matrix becomes mineralized, the formation of hydroxyapatite crystals generates large quantities of protons, which leads to extracellular acidification. In order to promote enamel maturation, ameloblasts act to neutralize the acidic environment by the concerted action of various acid–base transporters [1,3,27]. Consequently, local changes in pH may modulate both the uptake and secretion of Ca^2+^ by ameloblasts.

TRPM7 channels are known to be sensitive to changes in both intra- and extracellular pH [19,30]. In our experiments, extracellular acidification potentiated outward but not inward currents. In HEK-293 cells overexpressing TRPM7 channels, this treatment increased both outward and inward currents [19]. Since that study tested the effects of extracellular acidification in the absence of extracellular Mg^2+^, we suggest that the absence of proton-induced inward currents in HAT-7 cells was probably due to the presence of physiologic extracellular Mg^2+^ concentrations in our experiments.

It is tempting to speculate that the cyclical extracellular pH changes that occur during enamel formation could influence the uptake of Ca^2+^ via basolateral TRPM7 channels in the ameloblasts. Although we were unable to directly test the effects of changes in intracellular pH on channel function with electrophysiological methods, we did find that intracellular acidification of HAT-7 cells, using the ammonium pulse technique, markedly potentiated the Ca^2+^ influx induced by both naltriben and mibefradil. At first sight these data seem to contradict previous observations that, in the absence of intracellular Mg^2+^, cytosolic acidification inhibits the TRPM7 currents [30,44,45]. However, since protons and Mg^2+^ may share a common inhibitory site on the TRPM7 protein [30], we hypothesize that, in intact cells, intracellular acidification reduces the inhibitory effect of Mg^2+^, thereby activating the channel and inducing a Ca^2+^ influx. Whereas in previous patch-clamp studies, in which Mg^2+^ was omitted from the pipette solution, the binding of protons to the inhibitory site would have reduced any pre-existing TRPM7 channel activity.

**Figure 6:**
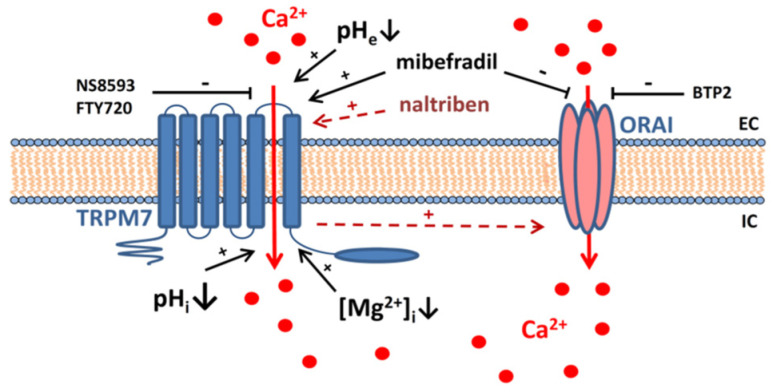
Suggested model for TRPM7-mediated Ca^2+^ entry in ameloblast-derived cells.

Taken together our results indicate the presence of functional TRPM7 channels in HAT-7 ameloblast cells that may serve as a Ca^2+^ uptake pathway sensitive to changes in both extra- and intracellular pH (Figure 6). TRPM7 may therefore play a role in the transport of divalent cations during amelogenesis. More specifically, we postulate that TRPM7 channels localized at the basolateral membrane of ameloblasts could provide a Ca^2+^ uptake pathway to supply Ca^2+^ for secretion across the apical membrane. Furthermore, these TRPM7 channels could be involved both as a positive modulator of the SOCE pathway, as reported previously [34], and as an independent, pH-sensitive Ca^2+^ uptake pathway, as we propose here.

## 4. Materials and Methods

### 4.1. Cell Culture

HAT-7 cells [21] were grown in Dulbecco’s modified Eagle’s medium and Nutrient Mixture F-12 Ham medium (DMEM:F12; Sigma Aldrich, St. Louis, MO, USA) supplemented with 10% HyClone characterised fetal bovine serum (FBS; Cytiva, Marlborough, MA, USA), 100 U/mL penicillin and 100 mg/mL streptomycin (Sigma Aldrich, St. Louis, MO, USA). Cells were maintained at 37 °C in 5% CO_2_ and passaged every 4 days with 0.25% trypsin-EDTA (Thermo Scientific, Waltham, MA, USA). Following activators and inhibitors were used in functional studies: naltriben (Tocris Bioscience, Bristol, UK), mibefradil (Tocris Bioscience, Bristol, UK), NS8593 (Tocris Bioscience, Bristol, UK), thapsigargin (Sigma Aldrich, St. Louis, MO, USA), FTY720 (Sigma Aldrich, St. Louis, MO, USA) and BTP2 (Merck KGaA, Dramstadt, Germany).

### 4.2. RT-qPCR

Total RNA was isolated from HAT-7 cells using a GeneJET RNA Purification Kit (Thermo Scientific, Waltham, MA, USA). The integrity of the RNA was assessed by running the purified RNAs on 1% agarose gels. Only samples in which the amount of 28S rRNA was about twice the intensity of the 18S rRNA were processed further. Total RNA (1 µg/sample) was reverse transcribed using a Maxima First Strand cDNA Synthesis Kit for RT-qPCR (Thermo Scientific, Waltham, MA, USA). Amplification was performed using the ABI StepOne System with TaqMan Universal Master Mix II (Applied Biosystems, Foster City, CA, USA) and predesigned primers (Life Technologies Magyarország Kft., Budapest, Hungary): TRPM7 (Rn01328216m1), Slc9a1/NHE1 (Rn00561924_m1), CFTR (Rn01455971_m1), Slc26a4/pendrin (Rn00693043_m1). Acidic ribosomal protein P0 (RPLP0; Rn00821065_g1) was used as internal control. Each sample was measured in three technical parallels. No-template and RT-minus reactions were performed to monitor nonspecific and genomic DNA amplifications, respectively. Relative fold changes were calculated by the comparative Ct method (2^−ΔΔ*C*T^).

### 4.3. Immunohistochemistry

HAT-7 cells were fixed with 95% ethanol/5% acetic acid. Hemimandibles from 6-week-old C57BL/6 mice were fixed with 4% paraformaldehyde, demineralized with 8% EDTA, embedded in paraffin and sectioned at 5 µm. After blocking with Trident Universal protein blocking reagent (GeneText, Irvine, CA, USA), slides were incubated with primary antibody at 4ºC overnight. The primary antibody was Abcam rabbit anti-TRPM7 antibody (ab262698; Abcam, Cambridge, UK) (dilution factor 1:300). Slides were then incubated with the secondary antibody (FITC-labelled goat anti-rabbit IgG, 1:400; Invitrogen, Carlsbad, CA, USA) at room temperature for 1 h. Nuclei were counterstained with 1 µg/mL Hoechst 33,342 (Sigma Aldrich, St. Louis, MO, USA) for 5 min at room temperature. Nonspecific rabbit IgG was used as the negative control. The slides were imaged using a Leica TCS SP5 confocal microscope (Leica Microsystems GmbH, Wien, Austria).

### 4.4. Electrophysiology

Voltage-clamp recordings were carried out in the standard whole-cell configuration using an Axopatch 200B amplifier (Molecular Devices, San José, CA, USA). Micropipettes were pulled with a P-97 Flaming-Brown-type micropipette puller (Sutter Instrument, Novato, CA, USA) from borosilicate glass capillary tubes (Harvard Apparatus, Cambridge, MA, USA) and had a tip resistance of 3–6 MΩ when filled with pipette solution. The intracellular solution contained (in mM): 120 CsCH_4_SO_4_; 20 NaCl; 10 HEPES; 2 EGTA; 0.9 MgCl_2_ pH 7.2 (with NaOH). In certain experimental protocols the Mg^2+^ content of the intracellular solution was either increased to 3.6 mM or reduced to ~0 mM (nominally Mg^2+^-free). The standard extracellular solution contained (in mM): 140 NaCl; 2.8 KCl; 1 CaCl_2_; 1 MgCl_2_; 10 HEPES; 11 D-glucose. The pH was adjusted to 7.3 with NaOH and to 4.3 with HCl. Solutions were delivered by continuous perfusion at 3 mL/min. Complete change of bath solution in the perfusion chamber required approximately 1 min (chamber volume ~1.5 mL). Effects on the currents were recorded at least 3 min after application of activators/inhibitors.

Whole-cell currents were measured at the holding potential (−50 mV) and during 30 ms square pulses of the test potential (−100 mV to +100 mV in 20 mV increments) at 0.5 s intervals. Before recording, currents were corrected for pipette capacitance, whole-cell capacitance, and series resistance. Current-voltage relations were based on stabilized currents measured 10 ms after applying the voltage pulses. Whole-cell currents were normalized to cell surface area as measured by whole-cell membrane capacitance (20 to 30 pF). Accordingly, currents are presented as pA/pF. Since currents measured at the holding potential in unstimulated conditions always remained below 0.5 pA/pF, we did not apply any leak current subtraction. The reversal potentials in our experiments ranged between −50 and +55 mV.

Command protocols and data acquisition were controlled by pClamp 11 software (Molecular Devices, San José, CA, USA). Capacitive currents were compensated by analog compensation. Series resistance was accepted if lower than five times the pipette tip resistance. Data were analyzed using Clampfit 11 (Molecular Devices, San José, CA, USA) and Microsoft Excel (Redmond, WA, USA) software. All experiments were performed at room temperature.

### 4.5. Calcium Imaging

Cells cultured on coverslips were incubated with 4 μM fura-2 AM (Invitrogen, Carlsbad, CA, USA) in bath solution for 45 min at room temperature and washed prior to calcium imaging. The coverslips were mounted on an upright fluorescence microscope (Nikon TE600, Nikon Europe BV, Amsterdam, Netherlands) in a custom-made open perfusion chamber and perfused with bath solution containing (in mM) 137 NaCl, 5 KCl, 2 CaCl_2_, 10 HEPES, and 10 glucose; pH 7.4 adjusted with NaOH. Mg^2+^ was omitted from the bath to avoid the inhibitory effect of Mg^2+^ on TRPM7 channel activity. In some experiments, a nominally Ca^2+^-free bath solution was used to investigate the source of the [Ca^2+^]_i_ responses. Cells were illuminated alternately with excitation wavelengths of 340 and 380 nm using a metal-halide lamp and an internal filter wheel (Prior Lumen 220 Pro, Prior Scientific, Cambridge, UK). Imaging was performed with a cooled CCD camera (QImaging Retiga2000; Teledyne Photometrics, Tucson, AZ, USA), coupled to the microscope and controlled by NIS AR software (Nikon Europe BV, Amsterdam, Netherlands). Changes in [Ca^2+^]_i_ were calculated as the ratio of the emitted fluorescence at the two respective excitation wavelengths (F340/F380) and normalized to the baseline ratio. Quantitative data were obtained by defining an ROI encompassing at least 50 cells.

### 4.6. Intracellular pH Measurements

Intracellular pH changes were monitored in real time by microfluorometry using the pH-sensitive fluorescent indicator BCECF as described previously [27]. Briefly, cells grown on coverslips were loaded with 4 µM BCECF-AM (Thermo Scientific, Waltham, MA, USA) for 30 min, then mounted in a chamber on a Nikon Eclipse TE200 inverted fluorescence microscope (Nikon Europe BV, Amsterdam, Netherlands) and superfused at 3 mL/min with the same bath solution as that used in the patch-clamp experiments. The pH-dependent (F490) and pH-independent (F440) fluorescence intensities were measured at 530 nm, with excitation alternating between 490 and 440 nm, using a photomultiplier tube and amplifier (Cairn Research, Faversham, Kent, UK). Data were acquired using DASYLab software (Measurement Computing, Norton, MA, USA) and the fluorescence ratio (F490/F440) was calculated at 5 s intervals. Fluorescence data were corrected for autofluorescence using Triton X-100 at the end of each experiment to release the intracellular BCECF. F490/F440 ratio values were converted to pH using calibration data obtained using the high K^+^/nigericin method [27].

### 4.7. Statistical Analysis

Data are presented as the mean ± SEM. Statistical analyses were performed with one-sample *t*-tests, planned pairwise comparisons using paired or unpaired two-sample *t*-tests as well as one-way ANOVA followed by Dunnett’s multiple comparison test; *p* < 0.05 was considered as significant.

## Data Availability

The data presented in this study are available in the article.
